# Rates and Determinants of Retention on ART Among Orphans and Vulnerable Children Living With HIV in Tanzania

**DOI:** 10.3389/fpubh.2022.934412

**Published:** 2022-07-28

**Authors:** John Charles, Amon Exavery, Amal Ally, Remmy Mseya, Tumainiel Mbwambo, Asheri Barankena, Christina Kyaruzi, Levina Kikoyo

**Affiliations:** Pact Tanzania, Dar es salaam, Tanzania

**Keywords:** retention, antiretroviral therapy, art, orphans, vulnerable, children, OVC, Tanzania

## Abstract

Despite the global progress in response to HIV and AIDS, notable challenges remain for children, especially identification, linkage, and retention in HIV care and treatment services. To succeed in pediatric HIV programming requires the linkage and retention of the children in those services over time. This study assessed the level of retention to antiretroviral therapy (ART) and its associated factors among orphans and vulnerable children living with HIV (OVCLHIV) in Tanzania. Data were obtained from the USAID Kizazi Kipya project that collected pediatric ART data from October 2017 to October 2019 in 81 district councils of Tanzania. Community-based volunteers supported the linkage and retention of the OVCLHIV on ART. Analysis of on-ART status was conducted in a cohort of OVCLHIV aged 0–20 years enrolled in the project and monitored for 24 months. OVCLHIV who remained on ART until the end of the follow-up period were referred to as “retained,” otherwise, “not retained”. Multivariable analysis was conducted using logistic regression, adjusting for baseline characteristics. Of the 5,304 OVCLHIV analyzed, the mean age was 13.1 years, 51.5% were female, and 72.2% were living with female caregivers. Their overall rate of retention on ART over the 24 months was 86.7%. Multivariable analysis showed that as the higher frequency of home visit by the project staff increased, the likelihood of retention increased by 8% [adjusted odds ratio (aOR) = 1.08, 95% CI 1.06–1.11, *p* < 0.001]. Membership in people living with HIV (PLHIV) support groups was associated with a higher likelihood of retention compared to nonmembership (aOR = 3.31, 95% CI 2.60–4.21, *p* < 0.001). Children in larger family size were 22% less likely to sustain ART (aOR = 0.78, 95% CI 0.72–0.84, *p* < 0.001). Urban OVCLHIV were 18% less likely to remain on ART than their rural counterparts (aOR = 0.82, 95% CI 0.69–0.98, *p* = 0.030). Remaining on ART was 49% more likely for OVC in economically better-off households than those in destitute households (aOR = 1.49, 95% CI 1.22–1.81, *p* < 0.001). Male OVC were 17% less likely to be retained on ART than their female counterparts (aOR = 0.83, 95% CI 0.71–0.99, *p* = 0.033). Community-based OVC support resulted in a high pediatric retention rate over the 24 months of follow-up. While key enablers of retention were higher frequency of home visits by the project volunteer, participation in PLHIV support groups, and better economic status, large family sizes, urban place of residence, and male gender of the OVC were barriers. This study brings useful evidence to inform strategies for advancing retention of OVCLHIV on ART for their better health outcomes and overall wellbeing.

## Introduction

Retention in HIV care and treatment services is key to successful prevention of disease progression and mortality among children living with HIV (CLHIV) (1, 2). In 2014, the UNAIDS launched the 90-90-90 goals for ending the AIDS epidemic, such that by 2020, 90% of all people living with HIV (PLHIV) are diagnosed, 90% of people diagnosed with HIV are initiated and sustained on antiretroviral therapy (ART), and 90% of people on ART are virally suppressed (3). The targets were later expanded to 95-95-95 for 2030 (4). To achieve these targets for pediatric population, CLHIV must be timely identified, linked to, initiated, and retained on ART to finally achieve viral load suppression (VLS) (5).

Due to the loss of one or both parents (6), orphans and vulnerable children (OVC) are not the same as other children in the general population. In areas such as sub-Saharan Africa where the effects of the HIV and AIDS are much felt, substantial evidence of the association between orphanhood and AIDS exists (7–9). OVC are more than twice more likely to have been infected with HIV by the time they reach adolescence than non-orphaned children (10, 11). Further evidence reveals that OVC are at a higher risk of being raised in poverty, dropping out of school, contracting HIV (12), as well as experiencing abuse and neglect (13). Considering the multiple vulnerabilities that OVC face, care and support prioritization are greatly required for this group for their ultimate wellbeing.

According to the UNAIDS, at the end of 2018, there were 92,000 children aged 0–14 years LHIV in Tanzania (14). Out of all these, only 65% were receiving treatment (14). Challenges in terms of coverage of care and treatment services for CLHIV remain, largely due to difficulties in identification, linkage, and retention (15). A recent study in Tanzania found a viral suppression rate of 65.8% among children and adolescents aged 0–19 years with a documented viral load test (16). This rate was low in comparison with the adult population (17) and the global target, suggesting possible issues with retention on, or adherence to, ART in this population.

Several factors influencing retention to treatment among CLHIV have been studied including but not limited to age (1, 2, 18), disease stage (1, 19), nutritional status (18, 20), and history of interrupted ART (21). However, most of these studies have been facility-based, lacking evidence of the role of community-based programs to support retention in HIV care and treatment services for PLHIV.

One longitudinal study from the Dar es Salaam region in Tanzania found among other things that there was a higher likelihood of loss to follow-up (LTFU) for CLHIV receiving treatment at a hospital instead of a local facility (18). This highlighted the need to assess the effectiveness of local- and community-based interventions to support retention into HIV care and AIDS response in general. Sustained ART and continued monitoring play an imperative role in achieving VLS and optimal treatment outcomes (1, 22). Interventions that are community-based to address gaps in linkage and retention on ART are greatly needed for addressing subpopulation-related barriers for sustained retention on ART. This study assessed the rates of retention on ART and explored OVC, caregiver, and household-level determinants of the retention of Tanzanian orphans and vulnerable children living with HIV (OVCLHIV) on ART.

## Materials and Methods

### Data Source and Study Area

Data for this study stem from a PEPFAR-funded OVC project known as USAID Kizazi Kipya which was implemented by Pact Tanzania for 5 years in almost all regions of Tanzania, from October 2016 to September 2021. The purpose of the project was to increase access to and uptake of HIV services as well as other health and social services by OVC and their household members. Enrollment into the project was voluntary, based on whether the household met one or more of the fourteen enrollment criteria that represent household vulnerabilities related to HIV as detailed elsewhere (23). A screening and enrollment tool was used to identify potential beneficiaries for the project. After enrollment, the family and child asset assessment (FCAA) tool was used to evaluate household needs for the purpose of developing a care plan. During household visits, services provided were tracked and captured using a monthly service tool, as well as a referral tool for referrals issued. Further details about the project, services provided, and tools completed are available (24).

Pediatric ART data used for this study were collected from October 2017 to October 2019 in 81 district councils in 25 regions of Tanzania. The district councils and the regions studied represented 44 and 81% of the country, respectively. Tanzania is administratively divided into regions, and each region is subdivided into districts. Then, each district is subdivided into divisions, which are further subdivided into wards, villages, and hamlets. Within hamlets are households. Lay community social welfare volunteers known as community caseworkers (CCWs) and lead caseworkers (LCWs) collected the data, conducted regular home visits, and supported linkage to and retention on ART for HIV-positive OVC. Each household enrolled in the project was visited by the LCWs or CCWs at least once every month. Households with more needs or challenges received more visits. Both LCWs and CCWs are all community-based volunteers who are trained to deliver the project services at the community level, including regular household visits. The former has additional roles to support the latter. Details of the cadres are provided in the respective standard operating procedures (25).

### Study Design and Population

This study was longitudinal in design, involving OVCLHIV on ART who were monitored for 24 months. The analysis of on-ART status was conducted on a cohort of 5,304 OVCLHIV aged 0–20 years who were enrolled in, and became beneficiaries of, the USAID Kizazi Kipya project in October 2017 and monitored for 24 months. Those whose ART status was not known were not included. In the PEPFAR context, an orphan and vulnerable child refers to a child aged 0–17 years who has either lost one or both parents or made more vulnerable because of HIV and AIDS (26). For implementation purpose, the USAID Kizazi Kipya extended up to 19 years to include all adolescents (25), and by the time this study was conducted, some had already turned 20 years.

### Variables

Retention on ART was the main outcome variable, defined as attending all care and treatment center (CTC) appointments by the OVCLHIV over the 24 months of follow-up. This was verified by the CCWs and LCWs during home visits by asking the caregiver or the OVC to report if they attended their last appointment, as well as following-up if they were adhering to ART. Where necessary, the CCW/LCW asked to see the OVCLHIV's CTC-1 card. This variable was organized as binary, with codes “1” and “0” for OVCLHIV who attended all their CTC appointments as scheduled and those who missed one or more appointments, respectively.

Several independent variables were included in this study including OVC age, OVC sex, OVC school enrollment status, membership in PLHIV groups, and the number of contacts by the project the child or caregiver received (i.e., frequency of home visit by CCWs). Others were caregiver sex, household size measured as the number of people living in the house, place of residence (rural or urban), and household economic status which was measured in the following two categories: destitute and well-off. Households in which some or all household members went whole day and night without eating anything three or more times in the last month were considered destitute and those that had rarely or never had this experience were considered well-off. This variable was derived from the question “In the past 4 weeks (30 days), did you or any household member go a whole day and night without eating anything because there was not enough food?” This question originates from the questions used in the construction of the Household Hunger Scale (HHS) by the Food and Agriculture Organization (FAO) and the Tufts University (27).

Regarding PLHIV groups, these bring together PLHIV and provide them with treatment support and literacy through the exchange of information and skills. In Tanzania, they consist of age-appropriate peer groups of children (age 0–14 years), adolescents (age 15–17 years), and adults (age ≥18 years). The groups support HIV status disclosure among other things, and for children and youth, caregivers may be involved to support their children's treatment. In PLHIV groups, children and adolescents play and have fun together, as well as normalize their experience of living with HIV (28). The groups were available for all children to attend. This variable was binary (yes/no), representing participation status of the OVCLHIV in the groups.

### Data Analysis

The data were summarized in frequencies to obtain descriptive statistics of the respondents through one-way tabulations as well as two-way tabulations. The one-way tabulations provided frequencies of each variable, while the two-way tabulations computed and compared the rates of retention on ART across different independent variables. In the latter case, the extent of association between retention and the independent variables, one at a time, was assessed using Pearson's chi-square test. Finally, a multivariable regression analysis of factors with an association with retention on ART was conducted using binary logistic regression while adjusting for baseline characteristics of the respondents. Statistical significance was inferred at 5% (α = 0.05). Analysis of the data was done using the Stata statistical software (version 14.0).

### Ethics Considerations

This study was conducted under the ethics approval numbered NIMR/HQ/R.8a/Vol.IX/3024 provided by the Medical Research Coordinating Committee (MRCC) of the National Institute for Medical Research (NIMR) in Tanzania. Participants were enrolled in the USAID Kizazi Kipya project only after their caregivers had consented and signed the consent form. All records and data of the project beneficiaries are stored securely and confidentially.

## Results

### Profile of Respondents

Of the 5,304 OVCLHIV included in this study, 51.4% were female and 71.4% lived with female caregivers; the mean age was 13.3 years. The majority (69.5%) were in economically destitute households. Slightly more than half (52.4%) were enrolled in school. A large majority (85.6%) were enrolled in PLHIV support groups, and 64.6% lived in rural areas (Table 1).

**Table 1:** Profile of orphans and vulnerable children (OVC) analyzed.

	**Number of** **respondents (*n*)**	**Percent of** **respondents (%)**
**All**	**5,304**	**100.0**
**OVC sex**
Female	2,724	51.4
Male	2,580	48.6
**Caregiver sex**
Female	3,786	71.4
Male	1,518	28.6
**Economic status**
Destitute	3,684	69.5
Well off	1,620	30.5
**Household size**
2 people	1,220	23.0
3 people	1,394	26.3
4 people	1,245	23.5
5 people	1,445	27.2
**School enrollment status**
Out of school	2,778	52.4
In school	2,526	47.6
**Attending PLHIV group**
No	763	14.4
Yes	4,541	85.6
**Place of residence**
Rural	3,425	64.6
Urban	1,879	35.4

### Retention by Background Characteristic

As shown in Table 2, the overall rate of retention on ART was 86.7% after 24 months of follow-up; however, the retention rate varied by different background characteristics. The retention rate was higher among OVC with male caregivers than those who had female caregivers (*p* = 0.019). With respect to household economic status, the retention rate was lower among OVC in destitute households than in those who were in economically well-off households (*p* < 0.001). The retention rate showed an inverse relationship with household size (*p* < 0.001) and it was also higher among OVC enrolled in school than those who were out of school (*p* < 0.001). Membership in groups of PLHIV was associated with a higher retention rate than that observed among nonmembers (*p* < 0.001). Finally, rural OVC's retention rate was significantly higher than urban OVCs (*p* < 0.001).

**Table 2:** Cross-tabulation of orphans and vulnerable children (OVC) living with HIV retained, and not retained on ART at the end of 24 months of follow-up by background characteristics (*n* = 5,304).

	**% (*n*)** **OVC retained**	**% (*n*) OVC** **not retained**	*p-*value*
**All**	**86.7 (4,599)**	**13.3 (705)**	**—**
**OVC sex**			0.062
Female	87.5 (2,384)	12.5 (341)	
Male	85.8 (2,214)	14.2 (366)	
**Caregiver sex**			0.019
Female	86.0 (3,256)	14.0 (530)	
Male	88.4 (1,342)	11.6 (176)	
**Economic status**			<0.001
Destitute	85.2 (3,139)	14.8 (545)	
Well off	90.0 (1,458)	10.0 (162)	
**Household size**			<0.001
2 people	90.7 (1,107)	9.3 (113)	
3 people	87.2 (1,216)	12.8 (178)	
4 people	85.6 (1,066)	14.4 (179)	
5 people or more	83.7 (1,209)	16.3 (236)	
**School enrollment status**			<0.001
Out of school	84.0 (2,334)	16.0 (444)	
In school	89.7 (2,266)	10.3 (260)	
**Attending PLHIV0 group**			<0.001
No	63.8 (487)	36.2 (276)	
Yes	90.5 (4,110)	9.5 (431)	
**Place of residence**			<0.001
Rural	88.1 (3,017)	11.9 (408)	
Urban	84.1 (1,580)	15.9 (299)	

^*^p-values are based on Pearson's chi-square test.

### Determinants of Retention

**Figure 1:**
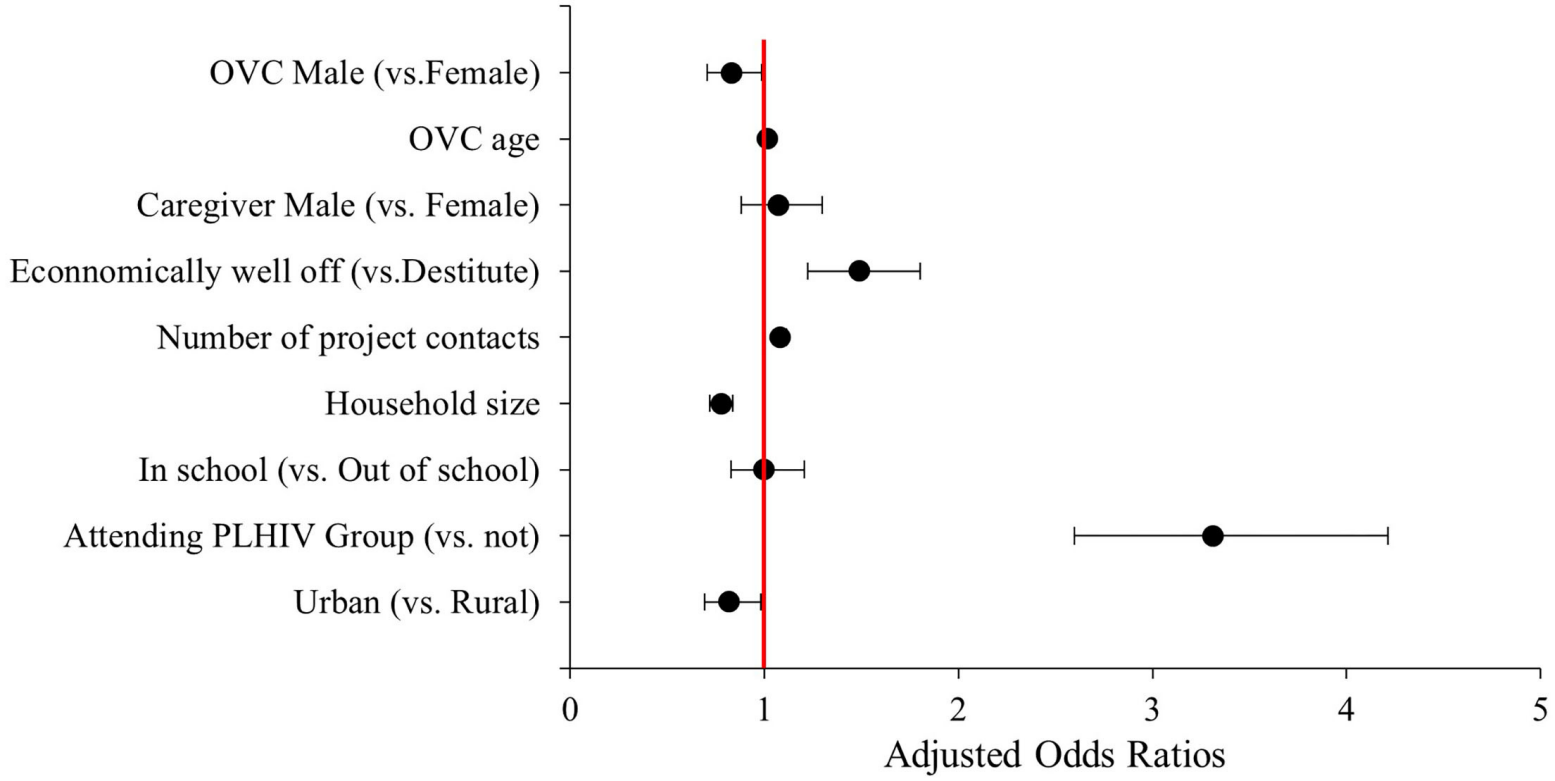
Multivariable logistic regression analysis of factors associated with 24 months retention on ART among orphans and vulnerable children (OVC) LHIV in Tanzania (*n* = 5,304). The factors were adjusted for OVC age and school enrollment status, as well as caregiver age and sex.

Multivariable analysis in Figure 1 presents adjusted odds ratios (aORs) and their corresponding 95% confidence intervals (CIs). The results revealed that male OVC were 17% less likely to be retained on ART than their female counterpart (aOR = 0.83, 95% CI 0.71–0.99, *p* = 0.033). A higher frequency of home visit by CCWs (i.e., project contact) increased the likelihood of retention on ART by 8% (aOR = 1.08, 95% CI 1.06–1.11, *p* < 0.001). Membership of HIV-positive children in HIV support groups improved the likelihood of retention on ART by more than three times (aOR = 3.31, 95% CI 2.60–4.21, *p* < 0.001). Children in larger family size were less likely by 22% to sustain ART over the 24 months period (aOR = 0.78, 95% CI 0.72–0.84, *p* < 0.001). OVCLHIV in urban area were 18% less likely to remain on ART than their rural counterparts (aOR = 0.82, 95% CI 0.69–0.98, *p* = 0.030). The likelihood of remaining on ART was 49% higher for OVC in economically better-off households than their peers in destitute families (aOR = 1.49, 95% CI 1.22–1.81, *p* < 0.001). These findings were adjusted for OVC age and school enrollment status, as well as caregiver age and sex.

## Discussion

This study assessed the rate of retention on ART among OVLHIV and its associated factors. As results revealed, the overall rate over the follow-up period was high and notably higher than that (72%) observed in a similar study which was health facility-based (1). The results also showed that participation in PLHIV support groups was the strongest determinant of retention on ART. Participation in PLHIV groups is beneficial and can also address stigma and discrimination, a factor that has been identified as one among the key barriers against retention in HIV services (29, 30). As already highlighted, HIV services that are pediatric- and adolescent-friendly have the potential to increase retention because beyond supporting HIV status disclosure, they reveal strategies for coping with the disease (31). All these contribute to treatment success through improved retention and adherence to ART for participants than nonparticipants, hence highlighting the importance of having all PLHIV in peer support groups. Community-based volunteers like the CCWs and LCWs are well-positioned to timely identify and address all surrounding barriers such as stigma, hesitancy, and myths during their home visits where they discuss with the OVCLHIV and their caregivers about pertinent matters related to their health and social wellbeing.

The study found that each one home visit by a trained project volunteer increased retention by 8%. Home visits by CCWs or LCWs involve the provision of direct services based on a personalized care plan such as psychosocial support, referrals, and tracking for services that the volunteer is not mandated to provide at the community level (e.g., referrals for viral load testing). Since the CCWs/LCWs are a trusted resident of the same community as the OVC families, she/he can easily detect and address any challenge or gaps in HIV treatment. Therefore, the OVC project has so far contributed to better retention through establishing, training, and supporting community-based CCWs/LCWs to scale up the coverage of HIV services in Tanzania.

In addition, the study observed that the likelihood of retention on ART was 49% higher among OVC in economically well-off families than their peers in destitute families. This was consistent with other studies (32, 33). It may be that due to caregivers' additional economic pressure in caring for OVC, they may be more likely to prioritize economic needs such as food availability in the home than consistently keeping their OVCLHIV in care and treatment services. This is supported by one study in Kenya that found better retention among children who were receiving food supplementation (20). Therefore, while economic empowerment interventions may be worthwhile for destitute families infected or affected by HIV, integrating food supplementation with HIV treatment services is likely to enhance retention and consequently improve treatment outcomes.

It was further observed that an increase in family size by one person resulted in a lower likelihood of retention on ART of the OVCLHIV over the follow-up period by 22%. Considering that the studied OVC are generally from poor families, it is likely that the bigger the family (for reasons such as multiple OVC cared for by the same caregiver), the higher the economic burden to the caregiver, hence, poor retention as discussed above. However, further research is needed to reveal the mechanism through which family size influences retention. Similarly, further studies should explain why retention was better in rural than in urban areas as well as in male than in female OVC.

## Limitations

People living with HIV support groups are health facility-based. This may require some participants to travel long distances and sometimes incur travel costs to get to where they are located. This may be a limiting factor for those who cannot afford the costs, hence influencing their retention. In addition, this study did not measure actual adherence to ART (i.e., pills taken per day). Due to the study design, the observed associations in this study may not be adequate for causal inferences.

## Conclusion

This study observed a high retention rate. Participation in PLHIV support groups was the most pervasive determinant of retention, suggesting a need to ensure that all OVCLHIV are in groups. OVC from economically well-off families were more likely to remain in treatment. Targeting destitute families with economic strengthening support is likely to enhance retention. In addition, OVC in larger families (3 or more people), and those residing in urban areas, may be at higher risk of being lost to the follow-up, hence a need to target them with additional support to keep them in care and treatment programs for better health outcomes among them.

## Data Availability Statement

The datasets analyzed for this study are confidential because they represent beneficiaries of a project. If necessary, they can be requested from Pact Tanzania. Requests to access these datasets should be directed to LK, lkikoyo@pactworld.org.

## Ethics Statement

The studies involving human participants were reviewed and approved by Medical Research Coordinating Committee (MRCC) of the National Institute for Medical Research (NIMR), Dar es Salaam, Tanzania. Written informed consent to participate in this study was provided by the participants' legal guardian/next of kin.

## Author Contributions

JC: problem conceptualization, study design, statistical analysis, drafting the manuscript, and critical review of the manuscript. AE: statistical analysis, literature review, drafting the manuscript, and review of subsequent versions. AA: data cleaning, analysis, and critical review of the manuscript for intellectual content. RM and TM: data management and critical review of the manuscript for intellectual content. AB, CK, and LK: critical review of the manuscript for intellectual content. All authors contributed to the article and approved the submitted version.

## Funding

The USAID Kizazi Kipya was a 5-year Project (from 2016 to 2021) implemented in Tanzania by Pact Tanzania as a prime organization, with funding from the U.S. President's Emergency Plan for AIDS Relief (PEPFAR) through the United States Agency for International Development (USAID) under the Cooperative Agreement No. (621-A-16-00001). The manuscript was produced as a part of the authors' employment functions.

## Author Disclaimer

The contents are the responsibility of the Pact and its project consortium partners and do not necessarily reflect the views of USAID or the United States Government.

## Conflict of Interest

The authors declare that the research was conducted in the absence of any commercial or financial relationships that could be construed as a potential conflict of interest.

## Publisher's Note

All claims expressed in this article are solely those of the authors and do not necessarily represent those of their affiliated organizations, or those of the publisher, the editors and the reviewers. Any product that may be evaluated in this article, or claim that may be made by its manufacturer, is not guaranteed or endorsed by the publisher.
